# The Influence of Single, Tandem, and Clustered DNA Damage on the Electronic Properties of the Double Helix: A Theoretical Study

**DOI:** 10.3390/molecules25143126

**Published:** 2020-07-08

**Authors:** Bolesław T. Karwowski

**Affiliations:** DNA Damage Laboratory of Food Science Department, Faculty of Pharmacy, Medical University of Lodz, ul. Muszynskiego 1, 90-151 Lodz, Poland; Boleslaw.Karwowski@umed.lodz.pl

**Keywords:** DNA damage, electronic properties, charge transfer, DFT, (5′*R*)/(5′*S*)-5′,8-cyclo-2′-deoxyadenosine, 8-oxo-7,8-dihydro-2′-deoxyguanosine

## Abstract

Oxidatively generated damage to DNA frequently appears in the human genome as the effect of aerobic metabolism or as the result of exposure to exogenous oxidizing agents, such as ionization radiation. In this paper, the electronic properties of single, tandem, and clustered DNA damage in comparison with native *ds*-DNA are discussed as a comparative analysis for the first time. A single lesion—8-oxo-7,8-dihydro-2′-deoxyguanosine (G^oxo^), a tandem lesion—(5′*S*) and (5′*R*) 5′,8-cyclo-2′-deoxyadenosine (cdA), and the presence of both of them in one helix turn as clustered DNA damage were chosen and taken into consideration. The lowest vertical and adiabatic potential (VIP ~ 5.9 and AIP ~ 5.5 eV, respectively) were found for G^oxo^, independently of the discussed DNA lesion type and their distribution within the double helix. Moreover, the VIP and AIP were assigned for *ds*-trimers, *ds*- dimers and single base pairs isolated from parental *ds*-hexamers in their neutral and cationic forms. The above results were confirmed by the charge and spin density population, which revealed that G^oxo^ can be considered as a cation radical point of destination independently of the DNA damage type (single, tandem, or clustered). Additionally, the different influences of cdA on the charge transfer rate were found and discussed in the context of tandem and clustered lesions. Because oligonucleotide lesions are effectively produced as a result of ionization factors, the presented data in this article might be valuable in developing a new scheme of anticancer radiotherapy efficiency.

## 1. Introduction

**Figure 1:**
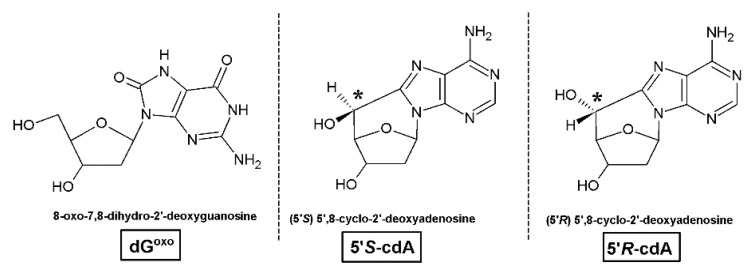
Graphical representation of the structure of the discussed DNA damage.

DNA is a storage house of genetic information in each cell of a living organism [1]. This information is continuously exposed to different kinds of harmful endo- and exogenous factors, such as ionization radiation (UV, gamma, X-ray), metabolic byproducts, etc. Their interaction with cellular oligonucleotides can cause the formation of DNA lesions both directly and indirectly. Until now, more than 70 types have been identified [2]. It is generally recognized that in the human body, 3 × 10^17^ DNA damage events per hour take place [3]. On the other hand, DNA lesions can be formed by the activity of the reactive oxygen/nitric species (ROS, RNS) [4]. It has been estimated that approximately 2 × 10^4^ free radical events per cell per day take place. Moreover, their number can increase with physical activity by up to 50% [5]. Of the plethora of radical oxygen species, the hydroxyl radical (^●^OH) has been found as the most reactive, with *k* = 2 − 10 × 10^−9^ M^−1^s^−1^ [6]. From the DNA damage distribution perspective, three main types of lesions can be distinguished: (a) Isolated—one lesion per one helix turn; (b) clustered—two or more per turn; and (c) tandem lesions as a result of a single DNA damage event in which a reactive nucleotide intermediate reacts with an adjacent one [7]. The DNA damage structures discussed in the article are shown in Figure 1.

Among all DNA lesions, 8-oxo-7,8-dihydro-2′-deoxyguanosine (dG^oxo^) is recognized as the most abundant. Its frequency in a cell has been estimated at 5.5 × 10^8^ [8,9]. At the other end of the scale, (5′*R*)/(5′*S*)-5′,8-cyclo-2′-deoxyadenosine ((5′*R*)-cdA and (5′*S*)-cdA) exist, and their frequency in cellular environments has been assigned as unequal 0.07/0.93 × 10^6^ *R*-cdA/*S*-cdA, respectively [10]. However, these results have yet to be verified scientifically and are still under discussion [11,12]. From the cellular point of view, these two diastereomers exhibit different biological/biochemical effects [13,14,15,16]. The stability of genetic information is crucial for the future generation of a species, and several repair systems are present in the cell, such as the base/nucleotide repair system (BER, NER), homologous and non-homologous end joining (HEJ, NHEJ), and nucleotide incision repair (NIR) [17]. Their correct activity guarantees the suitable nucleoside sequence in DNA. A failure, however, for example, in NER, can lead to different genetic disorders, cancer, or neurodegenerative disease [18,19,20].

For all the above repair processes to be effective, the recognition step is the most vital. The cascade of BER proteins starts from the glycosylases’ action. These enzymes can recognize and remove simple DNA damage, such as dG^oxo^, 2′-deoxyuridine [21]. To keep genetic material reproducible and stable, several specific glycosylases exist in cells, for example, OGG1 (8-oxo-guanine glycosylase 1), MutY (adenine DNA glycosylase), and UDG (uracil-DNA glycosylase) [22,23]. On the other hand, due to the additional C5′-C8 covalent bond, neither diastereomer of cdA is a substrate for the BER system—no cdA-specific glycosylases are known. The tandem lesions in both diastereomeric forms (5′*R*)-cdA and (5′*S*)-cdA are removed from the genome by the more complicated NER machinery. It is important to mention here that these small molecules, depending on the configuration on the 5′ carbon *S* or *R*, can significantly change the global structure of the DNA double helix, and as a result are removed from the genome at different rates [24]. The structure of the double helix and its changes are commonly described by a DNA standard reference frame. This analysis uses parameters that are useful for hydrogen bonding and base pairs’ stacking interaction description, which are fundamental for spatial DNA geometry pronunciation. For further details, please see the work of Olson et al. [25].

From an electronic point of view, *ds*-DNA can be perceived as a conductor of nanofibers [26], which has been shown by Shuster, Barton among others [27,28]. Recently, it was proposed that this phenomenon can allow MutY to scan the genome effectively with the electron transfer mode [29], even though the number of these protein copies is relatively low. MutY is able to verify/scan the *E. coli* genome (5 × 10^5^ base pairs) within 10 s [30]. For details, please see the review Barton et al. [31].

In this paper, comparative studies between isolated, clustered, and tandem DNA lesions, contained in (5′*R*)-cdA, (5′*S*)-cdA and dG^oxo^, and their influence on the electronic properties and charge transfer (CT) process of the double helix were considered. It is worth noting that little data exists in the literature that is dedicated to the influence of DNA damage on the hole transfer in *ds*-DNA [32,33,34].

## 2. Results and Discussion

To elucidate the influence of different types of DNA damage on charge transfer induced by a one-electron oxidizing event, nine double-stranded (*ds*) hexamers were chosen (Table 1).

The damage of interest was positioned in the central part of *ds-oigo*. The initial geometry of each in neutral and radical cation forms was optimized at the M062x/D95*:UFF level of theory in the aqueous phase using our own n-layered integrated molecular orbital and molecular mechanics (ONIOM) strategy [35]. The M062x functional was chosen as being suitable for estimating noncovalent interaction as well as for structural studies; additionally, the D95* basis set was used due to its efficiency in calculating such complicated systems in a reasonable time frame [36,37]. The electronic properties of the discussed *ds*-hexamers were obtained at the M062x/6-31+G** level of theory in the aqueous phase. However, due to the nature of DNA solvation, the aqueous phase relaxation influences the vertical ionization potential, and the vertical electron attachment was omitted [38]. This choice was sanctioned by the fact that the double helix is solvated from its outer and not internal shape where the base pair aromatic rings stack. The formed scaffold is the highway for hole migration via the hole hopping or super-exchange mechanism [39]. From the structural point of view, although the optimization of spatial geometry was performed for hexamers, only the central part (tetramer) was given further theoretical consideration. It is well known, and indeed observed in this study too, that the nucleoside pairs located on the 3′- and 5′-ends of *ds*-DNA adopted a deformed spatial structure on account of the lack of stacking interaction from one of the sides. Their inclusion in the discussion can obscure the clear and correct view of DNA electronic properties as well as charge transfer.

### 2.1. Structural Analysis of Isolated, Tandem, and Closured DNA Damage

The stability of the double helix depends on three factors: The hydrogen bond (HB) energies between complementary bases, the stacking energy within the base pair (BP) dimers, and solvation (first shape water layer) [40]. Although the mutual BP geometry is rather rigid and sensitive to structural changes (for example, crosslink, allylation, loss of bases, or part of the aromatic ring), the global spatial geometry of the double helix is to a greater or lesser extent similar. This phenomenon is derived from the high flexibility of the sugar-phosphate backbone, which keeps bases together in the oligonucleotide strands and prevents them from being scattered. Although the helix spine was taken for geometry optimization, due to its lack of significant meaning for hole transfer and electronic properties, it was removed and is not discussed further. The geometry analysis elucidated that G^oxo^ (the isolated lesion) appearing in the investigated double helix structures causes negligible h-rise parameter changes in comparison to the unmodified oligo, independently of its relative position to central A_3_. The h-rise parameters are presented in Table 2. The situation is different for the tandem lesion cdAs. These lesions forced h-rise increases, equal for both diastereomers, for the base pair dimers located on the 5′-end and 3′-end direction determined by cdA_3_. Subsequently, *h*-rise decreases between cdA_3_ and G_4_ were observed; however, a higher value was noted for (5′*S*)-cdA_3_ than (5′*R*)-cdA_3_, which can predict a different influence on the charge transfer process. These observations show that the rigidity of cdA cannot be eliminated by the sugar-phosphate backbone geometry rearmament in comparison to G^oxo^.

The second structural parameter that strongly influences the hole migration process is the mutual base pair spatial arrangement. It is worth noting that the aromatic ring overlap (ARO) can be perceived as the outcome of the tilt, twist, slide, shift, and roll standard DNA reference frame parameters. The following differences between native N-DNA and *ds*-oligo containing single, tandem or clustered lesions in the aromatic ring overlapping BP dimers were found (Table 3):

(a) The appearance of G^oxo^ in the discussed system leads to ARO decreases in all the investigated BP dimers except G_2_A_3_ of 3G^oxo^-N-DNA. The above indicates by comparison with 5G^oxo^-N-DNA that G^oxo^ forces BP flipping from the ideal/parent position in its 3′- and 5′-end directions.

(b) The tandem lesion appearing in the *ds*-oligonucleotide, i.e., *R*- or *S*- cdA, far more strongly disrupts the double helix structure than G^oxo^. Surprisingly, the presence of (5′*R*) or (5′*S*) 5′,8-cyclo-2′-deoxyadenosine leads to G_4_G_5_ aromatic ring overlapping increases by −1.6 Å^2^. On the other hand, (5′*R*)-cdA affected ARO in the case of the cA_3_G_4_ base pair dimer more strongly than (5′*S*)-cdA, with the following values found [in Å^2^]: 0.69 and 1.79 for ScA-DNA and RcA-DNA, respectively. These differences indicate that (5′*R*)-cdA disrupts more strongly the spatial *ds*-DNA structure than the opposite diastereomer, and therefore the effect of this difference should be visible in the values of the charge transfer process parameters.

(c) The geometrical analysis of the clustered lesion, composed of cdA and G^oxo^, reveals that the (5′*S*)-cdA causes ARO increases independently of the relative G^oxo^ 3′ or 5′-end position of cdA. The situation is different in the case of (5′*R*)-cdA if G^oxo^ is present at the 3′ hydroxyl group site of cdA with a BPs ARO decrease being observed. Contrary to the above, G^oxo^ shifted to the 5′ site of (5′*R*)-cdA, causing aromatic ring overlapping increases within the A_3_G_4_ base pair dimers. Based on the above, it can be expected that in the case of clustered lesions, (5′*R*)-cdA should more strongly affect hole migration than the opposite diastereomer.

The *h*-rise parameter and ARO are strongly connected with stacking interaction (ST) and have a strong influence on it. This non-covalent interaction is the second force that stabilizes the double helix. The thorough stacking energy analysis (see Table 4) between BP involved directly in the dimer structure shows that the presence of G^oxo^ leads to stacking energy increases within dimers A_2_G_4_ and G_4_G_5_. Surprisingly, when G^oxo^ is shifted to the G_2_ position, decreases in ST energy of G_2_A_3_ and A_3_G_4_ were observed. Similar results were found for all the discussed tandem and clustered lesions, except one, i.e., a stacking interaction energy increase was observed for G_2_A_3_, G_4_G_5_ BP dimers, with a subsequent decrease in the case of G_4_G_5_. The presence of G^oxo^ on the 3′-end site of R-cdA (3G^oxo^-RcA-DNA) leads to opposite results, with an ST energy decrease noted for the G_2_cA_3_ dimer, while for the remaining two, rises in its value were assigned. The above results strongly indicate that the influence of DNA damage on stacking interaction strongly depends on the oligonucleotide base sequence. However, it should be pointed out that for all the discussed DNA lesions, the ST energy increases in the G_4_G_5_ dimer were observed in a range between 0.04 and 2.79 kcal/mol. These observations indicate that the hole transfer between GG is preferred, which is in good agreement with previous theoretical and experimental data [41].

As mentioned above, the stability of the DNA double helix is the result of stacking and hydrogen bond energies. From previous studies, it is known that base modification and DNA damage can strongly affect mutual complementary base interaction and therefore influence HB energy [34,42]. The results discussed below are presented in Table 5. The comparison of *ds*-DNA containing a single or clustered lesion with native DNA elucidated that the dG^oxo^ appearing in the oligonucleotide causes increases in the HB energy of the dC:::dG^oxo^ pair independently of other lesions present in the range between 0.38 and 0.87 kcal/mol. For a single DNA lesion, the HB energy increases in the dC:::dG^oxo^ pair were almost the same for 3G^oxo^-N-DNA and 5G^oxo^-N-DNA, i.e., 0.46 and 0.54 kcal/mol, respectively. Moreover, for oligonucleotides containing only dG^oxo^, the HB energies calculated for other base pairs were almost unaffected in comparison with N-DNA, except the A_3_::T_3_ base pair of 3G^oxo^-N-DNA, for which a fall of 0.28 kcal/mol was noted. Opposite results were noted for other *ds*-DNA with tandem or clustered lesions in all cases other than for dC::dG^oxo^ base pairs, where a decrease in HB energy was observed. The results presented above indicated that clustered or tandem lesions strongly affect the double helix structure and influence its stability. The experimental data shows that (5′*S*)-cdA leads to melting temperature value decreases of 6 °C [43], while dG^oxo^ affected these parameters only negligibly by 2 °C [44] in comparison to unmodified *ds*-oligo.

### 2.2. The Ionization Potential of Isolated, Tandem, and Closured DNA Damage

**Figure 2:**
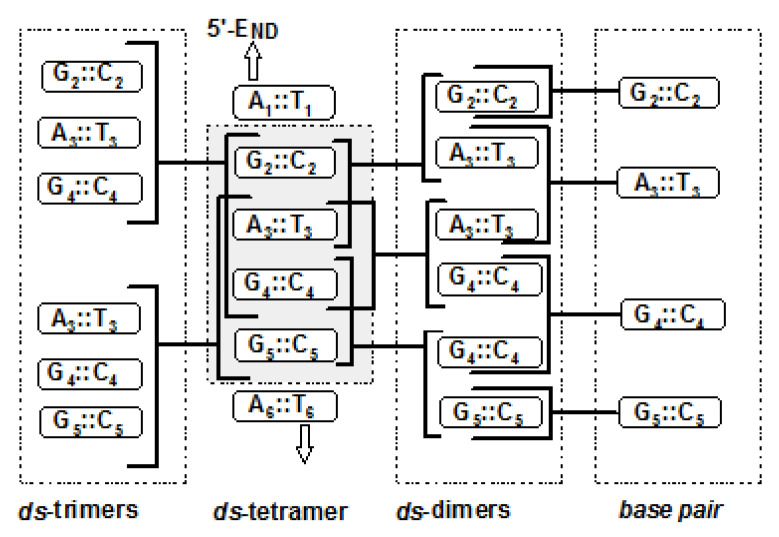
Graphical representation of *ds*-oligonucleotides divided into two *ds*-trimers, three *ds*-dimers, and four base pairs (indicated by dashed squares).

During the genome one-electron oxidation process initiated by, for example, exposure to ionization radiation, radical cations can be formed randomly. The holes (radical cations) can migrate and become trapped at some preferred places of the oligonucleotide structure with the lowest ionization potential. The following order of nucleic base ionization potential (IP) has been noted: thymine ≈ cytosine > adenine > guanine; additionally, the following radical distribution has been noted during oligonucleotide γ-radiation: 35% G^●+^, 5% A^●+^, and about 45% of T^●−^ and C^●−^ [47]. Therefore, it can be concluded that pyrimidines have a higher ionization potential than purines. Moreover, 8-oxo-2′-deoxyguanine has a lower ionization potential than dG and is easiest to oxidize [48]. The above results are in good agreement with those presented in this article. Table 6 presents the adiabatic/vertical ionization potential of the isolated base, with base pairs calculated at the M062x/6-31+G** level of theory in the aqueous phase. The situation is a little bit more complicated when *ds*-DAN is taken into consideration. Independently, Senthilkumar and Voityuk calculated the vertical ionization potential (VIP) of all double-stranded tetramers [49,50]. From their study it is clear that the VIP of the tetramers depends on their sequences. In these studies, both the adiabatic and vertical IP of trimers contained within the tetramer structures (extracted as a central part of optimized *ds*-hexamers: Figure 2), as well as *ds*-dimers and isolated base pairs, were taken into theoretical investigation. In all the investigated *ds*-trimers, the lowest VIP and AIP values were found for (3G^oxo^-N-DNA) A_3_^oxo^G_4_G_5_ (5,37/5,79 eV), which are lower by 0.06 and 0.14 eV, respectively, than those noted for ^oxo^G_2_A_3_G_4_. It is important to mention that the lack of oxidized guanosine in the structure eliminates the difference between the vertical and adiabatic state as observed for A_3_G_4_G_5_ extracted from 5G^oxo^-N-DNA. The VIP of this trimer was found at the same level as that of the corresponding one in N-DNA (VIP = 6.02 eV). As presented in Table 6, the same pattern of VIP and AIP was observed for the discussed tandem and clustered DNA lesions. It is important to mention that the presence of both cdA diastereomers causes slight ionization potential increases in clustered and tandem DNA damage in comparison to the corresponding trimers of native or single-lesioned *ds*-oligo in the following range: VIP: 0.01–0.12 eV and AIP: 0.02–0.05 eV. Based on the above, it can be postulated that G^oxo^ is a crucial factor, which determines the sink of radical cations and is able to cover the cdA structural influence on the electronic properties of *ds*-trimers.

The investigated *ds*-tetramers divided into three base pairs *ds*-dimers show that in the structure of native *ds*-DNA, the lowest VIP and AIP were noted for G_4_G_5_ (6.05/5.68eV), which was expected. Only negligible differences between the adiabatic and vertical ionization potential were found for the G_2_A_3_ moiety. The situation was similar in the case of 3G^oxo^-N-DNA (single-lesioned *ds*-oligo), in which the ^oxo^G_3_G_4_ part becomes a hole trap. The 8-oxo-2′-deoxyguanosine shift into the G_2_ position changes the pattern of IP distribution. The lowest VIP and AIP was noted for ^oxo^G_2_A_3_ *ds*-dimer (5.91/5.50 eV), while for other *ds*-dimers, a difference between IPs was not observed. The corresponding results were found for clustered DNA damage in which A_3_ was changed by (5′*R*)- or (5′*S*)-cdA. It should be pointed out that in all the above-discussed cases, the lowest calculated value of the vertical ionization potential, among the isolated dimers, corresponds to the lowest adiabatic IP. The situation is the opposite in the case of the tandem lesion: A discrepancy between VIP and AIP was noted. The lowest VIP was found for the G_2_cA_3_ *ds*-dimer of ScA-DNA and the cA_3_G_4_ of RcA-DNA, while the lowest AIP was calculated for A_3_G_4_ and G_4_G_5_, respectively (Table 6). These observations indicate that cdA appearing in the double helix leads to structural changes, which can obscure the charge migration process. As focused on in Table 3, the geometry rearrangement and its energetical pronunciation (Table 4 and Table 5) are more visible after one-electron oxidation. To confirm the above results, parent *ds*-tetramers were divided into four single base pairs for which the VIP and AIP were calculated at the M062x/6-31+G** level of theory in the aqueous phase. The obtained results show that in the case of dG^oxo^ being absent, the lowest VIP and AIP were assigned for G_4_C_4_ BP independently of which *ds*-tetramer (native, single, tandem, or cluster lesioned) was isolated. As can be expected for the rest of the discussed *ds*-oligos, the lowest value of vertical and adiabatic IP was found for base pairs that contained G^oxo^ in their moiety. Moreover, almost the same values of these parameters were noted for G_4_C_4_ and ^oxo^G_3_C_3_: 6.1/5.8eV and 5.9/5.5 eV VIP /AIP, respectively. These results are in good agreement with the experimental data, which shows that the 5′-end GC pair in the d[GG]*d[CC] dimer is the most easily oxidized (due to it having the lowest VIP and AIP) [51]**.** Additionally, in each discussed case, ^oxo^GC BP had a lower VIP and AIP by 0.3 eV than the parent GC pair as shown in Table 6. For the remaining base pairs, the assigned IP values fluctuated. However, what is surprising is that the VIP was mainly noted as lower or at the same level as the adiabatic IP. Based on an ionization potential and structural analysis, it can be concluded that the hole migrated through *ds*-DNA without each BP structural rearrangement, which is necessary for the VIP→AIP conversion. Therefore, the hole slides through the double helix until it settles in the ”pleasant” part of *ds*-oligo, thanks to it having the lowest VIP and AIP. 

A comparative spatial geometry analysis of the discussed *ds*-tetramer between their initial geometry of adiabatic neutral and positively charged states shows that native N-DNA and 5G^oxo^-RcA-DNA are the most sensitive to adiabatic radical cation formation (Table 7). In other cases, the presence of dG^oxo^ or cdA eliminate the structural changes forced by electron loss of *ds*-oligo. It can be predicted that DNA damage formation makes the hole transfer process much easier towards the radical cation sink formed by dG^oxo^ than in the case of unmodified *ds*-oligo, which required significant double helix changes for positive charge compensation. The rigidity of (5′*R*/5′*S*)-cdA (tandem lesion) makes the *ds*-DNA structure resistant to positive ionization. Additionally, the appearance of clustered damage formed by cdA and dG^oxo^ in the case of 5G^oxo^-RcA-DNA leads to significant geometry changes (Table 7) in comparison to others.

In the ionization potential analysis presented above, differences between the discussed *ds*-oligo were observed forcing the comprehensive charge and spin analysis, presented in Table 8. As expected, independently of the type of *ds*-oligo, whether undamaged, isolated, tandem, or clustered lesioned, the charge and spin are mainly located on G^oxo^ or the 5′G_4_ of the G_4_G_5_ dimer in each case. Moreover, the difference between the vertical and adiabatic radical cation form of *ds*-DNA was negligible. These observations confirm the results (ionization potential) discussed above, which indicate that independently of the system after a complete dismantle of *ds*-DNAs into constituent base pairs, G^oxo^ or 5′G_4_ of G_4_G_5_ can be considered a suitable part of the double helix for positive charge accumulation.

As mentioned above, the charge transfer through the double helix independently of the damage type can be described as a super-exchange or multistep hopping process [28,34]. Using the previously described strategy, the barrier (Δ*G*) for hole transfer within interlaced trimers was assigned in vertical and adiabatic modes (Figure 2, Table 9) [42]. It was found that in all the discussed *ds*-oligonucleotides, the “hole” appearing in the double-helix structure preferably migrated to G_4_ or G^oxo^ independently of its position in the *ds*-DNA. These results are in excellent agreement with the experimental data obtained by Schuster [41].

The charge transfer migration through the double helix can be described according to the Marcus theory, which states that the rate constant (*k*_ET_) of charge transfer (CT) depends on several factors: The structure of π-stacks, i.e., BPs, the driving force (Δ*F*), nuclear reorganization (λ), activation (*E*_a_) and the electron-coupling (*V*_12_) energies [52]. *V*_12_ was calculated according to the GMH (generalized Mulliken–Hush) strategy within the terms of the occupied Kohn–Sham orbital method [53,54]. The charge transfer, which passes through the adiabatic states of the donor and acceptor, is associated with the movement of internal geometries (atoms), expressed by λ in the Marcus theory. All the above parameters calculated for the systems discussed in this article are presented in Table 10.

An analysis of the reorganization energies reveals a significant rise in the A_3_G_4_, G_4_G_5_ dimer in the case of native unmodified N-DAN and for cdA_3_G_4_, G4G4 of *ds*-DNA containing a tandem lesion. Moreover, the same was noted when G_4_ was converted into G^oxo^ (3G^oxo^-N-DNA, 3G^oxo^-RcA-DNA, 3G^oxo^-ScA-DNA). It is important to mention that in the case of damage being present in the double helix, the reorganization energy of the A_3_G_4_ dimer is almost equal to that found for G_4_G_5_, while for unmodified *ds*-oligo (N-DNA), the λ of G_4_G_5_ was two times higher than for A_3_G_4_. For the oligonucleotides where G^oxo^ changed to the G_2_ position, the highest λ was denoted for the G_2_^oxo^A_3_ dimer (approximately 0.40 eV) while for the remaining dimers, the value was significantly lower (0.01–0.04 eV). However, for 5G^oxo^-RcA-DNA, the λ of A_3_G_4_ and G_4_G_5_ should be noted as follows: 0.16 and 0.12 eV respectively. This strongly indicates that G^oxo^ plays an invaluable role in genome protection, taking the role of radical slope/trash instead of both diastereomers of cdA (tandem lesion). Due to the fact that *k*_HT_ is strongly dependent on the distance and aromatic ring overlapping between the donor and acceptor, an influence of the single, tandem, and clustered DNA lesion on charge transfer in the double helix shape can be expected in comparison with unmodified *ds*-DAN. Table 9 presents the discussed parameters of the charge transfer process.

The calculated *k*_HT_s value between the base pair dimers of the reference *ds-oligo* gives the following values: 0.00 for **G_2_←**A3, 3.8 × 10^11^ for **A_3_→G_4_** 4.0 × 10^13^, and **G_4_←G_5_**. The obtained higher value for G_4_←G_5_ is in good agreement with recent theoretical studies, which have postulated that the hole migrated in the 5′-end direction of GG dimers [32]. The single lesion formation in the double helix influence on the CT process depends on its place of settlement. The presence of G^oxo^ as part of the G_4_G_5_ dimer (on its 5′-end) leads to a greater CT rate increase by one order of magnitude than for N-DNA, with subsequent significant *k*_HT_ decreases for A_3_→G_4_ transfer (Table 9). The G^oxo^ shift to the G_2_ position causes the CT rate to increase between A_3_→G_2′_ in comparison to native DNA up to 3.2 × 10^10^. However, the *k*_HT_ assigned for G_4_→G_5_ transfer was at the same level as observed for N-DNA while ΔG decreases were noted as well.

The formation of 5′,8-cyclo-2′-deoxyadenosine in the double helix leads to different results depending on the C5′ chirality. The (5′*S*)-cdA force the same effect as discussed for a native *ds*-oligo (N-DNA), when it has been considered as a tandem lesion. The configuration inversion on the C5′ of cdA forces *k*_HT_s increases in all the discussed CTs ((5′R)cA_3_→G_2_; (5′*R*)cA_3_→G_4_; G_5_→G_4_) (Table 9). These observations indicate that the charge transfer within the double helix can be disturbed by structural changes forced by (5′*R*)-cdA (Table 2, Table 3 and Table 4). Moreover, based on the energy barrier analysis presented in Table 9, the transfer between G_2_→G_4_ can take place in the adiabatic mode (−0.33 eV). More details on calculated energy levels can be found in the Supplementary Materials.

The presence of G^oxo^ and (5′*R*)- or (5′*S*)-cdA in the same helix turn leads to clustered damage formation. In the case when G^oxo^ is part of the ^oxo^G_4_G_5_ dimer (3G^oxo^-ScA-DNA and 3G^oxo^-RcA-DNA), the charge transfer is allowed for cA_3_→G_4_^oxo^ and G_5_→G_4_^oxo^. However, (5′*S*)-cdA increases the hole migration from (5′*S*)cA_3_→G_4_ by one order of magnitude in comparison with N-DNA, while for the opposite diastereomer, this value was two orders of magnitude higher. Subsequently, (5′*S*)-cdA left *k*_ET_ of G_5_→G_4_^oxo^ at the same level as was assigned for native N-DNA for (5′*R*)-cdA; this value was found to be one order of magnitude lower. The above indicates that depending on the C5′ chirality, cdA can modulate the charge transfer in its 3′- or 5′-end direction in the case of a clustered DNA lesion. This was confirmed by the results obtained for 5G^oxo^-ScA-DNA and 5G^oxo^-RcA-DNA, where G^oxo^ was shifted to the G_2_ position (Figure 2, Table 1). As presented in Table 10, the presence of (5′*S*)-cdA slows down the (5′*S*)cA→G_2_^oxo^ charge transfer by three orders of magnitude, while (5′*R*)-cdA is only by two in comparison with 5G^oxo^-N-DNA. Subsequently, both cdA diastereomers left G_5_→G_4_ at the same level as forced by dA in suitable single-lesioned DNA. Surprisingly, in the case of 5G^oxo^-RcA-DNA, the (5′*R*)cA_3_→G_4_ was found to be allowed/possible—*k*_ET_ = 1.75 × 10^8^—and was at the same level as that assigned for (5′*R*)cA_3_→G_2_^oxo^, which indicates that the 5′*R* diastereomer is able to disturb the charge transfer process. These observations are in good agreement with previous theoretical studies in which the directional effect of cdAs was noted [34].

## 3. Materials and Methods

### 3.1. Computation Methodology of QM/MM Studies [42,43]

The geometry optimizations of *ds*-hexamers presented in Table 1 were performed using the QM/MM strategy [35,36]. The structures of the double-stranded oligonucleotides were divided into high- HL (nucleobases, M06-2X/D95*), and low- LL (sugar-phosphate backbone, UFF) levels of calculation using ONIOM in the aqueous phase [55]. The solvent effect was described for an aqueous medium, applying Tomasi’s polarized continuum model [56]. The negative charges of each phosphate group were neutralized by the addition of protons. The full structure optimized *ds*-hexamers were converted to base pairs by sugar-phosphate backbone removal. In the formed base pair systems, the atoms were saturated, if necessary, with hydrogen atoms. The spatial location of the hydrogen atoms added for saturation were optimized at the M06-2X/D95* level of theory in the aqueous phase, with the position of all other atoms frozen.

### 3.2. Computation Methodology of Density Functional Theory (DFT) Study

All energy calculations were performed in the aqueous phase by the density functional theory (DFT) using the M06-2X functional with a double-ζ 6-31+G** basis set [57,58]. The characterization of the transition dipole moment of excited states and the single point calculation at the M06-2X/6-31+G** level of theory were performed using time-dependent DFT (TD-DFT) methodology [59]. For all the optimized structures, a charge and spin analysis was achieved using Hirshfeld methodology at the M06-2X/6-31+G** level [60]. The electron coupling was calculated using generalized Mulliken–Hush methodology [61]. The electronic properties were calculated as previously described [62]. All calculations were performed in the aqueous phase with the Gaussian 09 (revision A.02) software package [63]. The three-dimensional structural analyses of the mentioned ss- and *ds*-DNAs, based on a standard reference frame, were obtained with by a 3DNA software package using the web-based interface w3DNA (web 3DNA) [64].

## 4. Conclusions

The appearance of different types of single, tandem, or clustered DNA lesions in the oligonucleotide sequence gives rise to various consequences of charge transfer in comparison with native *ds*-oligo (N-DNA). In this article, for the first time, a comparative analysis was made between unmodified *ds*-oligo and one which contains G^oxo^, cdA, or both. Both types of lesions taken into consideration can be formed by hydroxyl radical activity. However, the dG^oxo^ by •OH addition to the C8 moiety of dG while the 5′*R* and 5′*S* diastereomers of 5′,8-cyclo-2′deoxyadenosine can occur in *ds*-DNA as a result of a two-step cyclization reaction induced by hydrogen atom abstraction from the C5′ position by a hydrogen radical [65]. These unusual tandem lesions can lead to different local spatial geometry changes in the double helix, next to their place of formation [24]. Probably, as a result, (5′*R*)-cdA and (5′*S*)-cdA(S) had a disparate influence on BER enzyme activities, as well as on the electronic properties of the *ds*-DNA part, next to its appearance. The results presented above indicate that dependent on C5′ chirality, cdA can modulate the charge transfer toward its 3′- or 5′-end direction in the case of a clustered DNA lesion. However, in all the discussed DNA lesions, the appearance of dG^oxo^ in the double helix structure constitutes the final destination of radical cation migration.

## Figures and Tables

**Table 1:** Nucleobase sequence “structures” of double-stranded oligonucleotides taken into theoretical consideration. ^oxo^G - 8-oxo-7,8-dihydro-2′-deoxyguaosie, (5′*S*)-cA: (5′*S*)-5′,8-cyclo-2′-deoxyadenosine, (5′*R*)-cA: (5′*R*)-5′,8-cyclo-2′-deoxyadenosine.

DNA Damage Type	Oligonucleotide	Oligonucleotide Base Sequence
Undamaged Native *ds*-DNA	N-DNA	d[A_1_G_2_A_3_G_4_G_5_A_6_]*d[T_6_C_5_C_4_A_3_C_2_T_1_]
Single	3G^oxo^-N-DNA	d[A_1_G_2_A_3_^oxo^G_4_G_5_A_6_]*d[T_6_C_5_C_4_A_3_C_2_T_1_]
	5G^oxo^-N-DNA	d[A_1_^oxo^G_2_A_3_G_4_G_5_A_6_]*d[T_6_C_5_C_4_A_3_C_2_T_1_]
Tandem	ScA-DNA	d[A_1_G_2_(5′S)cA_3_G_4_G_5_A_6_]*d[T_6_C_5_C_4_A_3_C_2_T_1_]
Clustered	3G^oxo^-ScA-DNA	d[A_1_G_2_(5′S)cA_3_^oxo^G_4_G_5_A_6_]*d[T_6_C_5_C_4_A_3_C_2_T_1_]
	5G^oxo^-ScA-DNA	d[A_1_^oxo^G_2_(5′S)cA_3_G_4_G_5_A_6_]*d[T_6_C_5_C_4_A_3_C_2_T_1_]
Tandem	RcA-DNA	d[A_1_G_2_(5′R)cA_3_G_4_G_5_A_6_]*d[T_6_C_5_C_4_A_3_C_2_T_1_]
Clustered	3G^oxo^-RcA-DNA	d[A_1_G_2_(5′R)cA_3_^oxo^G_4_G_5_A_6_]*d[T_6_C_5_C_4_A_3_C_2_T_1_]
	5G^oxo^-RcA-DNA	d[A_1_^oxo^G_2_(5′R)cA_3_G_4_G_5_A_6_]*d[T_6_C_5_C_4_A_3_C_2_T_1_]

**Table 2:** The *h*-rise parameters of base pair dimers obtained for the discussed *ds*-oligonucleotides in their neutral (Neut.) and adiabatic radical cation (ARC) form geometries.

*ds*-DNA Base Dimer *h*-rise Parameter [Å]								
**N-DNA**			**3G^oxo^-N-DNA**			**5G^oxo^-N-DNA**		
	**NEUT.**	**ARC**		**NEUT.**	**ARC**		**NEUT.**	**ARC**
G_2_A_3_	2.96	3.01	G_2_A_3_	3.06	3.02	^oxo^G_2_A_3_	2.89	2.86
A_3_G_4_	3.31	2.88	A_3_G_4_^oxo^	3.33	3.25	A_3_G_4_	3.24	3.23
G_4_G_5_	3.34	3.14	^oxo^G_4_G_5_	3.28	3.07	G_4_G_5_	3.34	3.38
**ScA-DNA**			**3G^oxo^-ScA-DNA**			**5G^oxo^-ScA-DNA**		
G_2_(5′*S*)cA_3_	3.36	3.29	G_2_(5′*S*)cA_3_	3.36	3.39	^oxo^G_2_(5′*S*)cA_3_	3.26	3.11
(5′*S*)cA_3_G_4_	2.98	2.82	(5′*S*)cA_3_G_4_^oxo^	3.05	2.87	(5′*S*)cA_3_G_4_	2.94	2.97
G_4_G_5_	3.68	3.56	^oxo^G_4_G_5_	3.65	3.5	G_4_G_5_	3.68	3.68
**RcA-DNA**			**3G^oxo^-RcA-DNA**			**5G^oxo^-RcA-DNA**		
G_2_(5′*R*)cA_3_	3.32	3.26	G_2_(5′*R*)c A_3_	3.45	3.41	^oxo^G_2_(5′*R*)c A_3_	3.37	3.24
(5′*R*)cA_3_G_4_	2.8	2.7	(5′*R*)cA_3_G_4_^oxo^	3.15	3.12	(5′*R*)cA_3_G_4_	2.87	3.05
G_4_G_5_	3.6	3.49	^oxo^G_4_G_5_	3.68	3.55	G_4_G_5_	3.63	3.67

**Table 3:** Aromatic ring overlapping of the base pair dimers of the discussed *ds*-oligonucleotides in their neutral (Neut.) and adiabatic radical cation (ARC) form geometries.

*ds*-DNA Bases Aromatic Rings Overlap [Å^2^]								
**N-DNA**			**3G^oxo^-N-DNA**			**5G^oxo^-N-DNA**		
	**NEUT.**	**ARC**		**NEUT.**	**ARC**		**NEUT.**	**ARC**
G_2_A_3_	2.14	1.3	G_2_A_3_	2.95	2.9	G_2_^oxo^A_3_	1.95	1.62
A_3_G_4_	3.79	3.46	A_3_G_4_^oxo^	2.56	2.93	A_3_G_4_	3.31	3.66
G_4_G_5_	1.28	1.07	G_4_^oxo^G_5_	0.56	0.52	G_4_G_5_	0.77	0.83
**ScA-DNA**			**3G^oxo^-ScA-DNA**			**5G^oxo^-ScA-DNA**		
G_2_A_3_	2.29	2.2	G_2_A_3_	2.27	2.22	^oxo^G_2_A_3_	1.95	2.14
(5′*S*)cA_3_G_4_	3.10	5.59	(5′*S*)cA_3_G_4_^oxo^	6.51	6.31	(5′*S*)cA_3_G_4_	5.30	5.21
^oxo^G_4_G_5_	2.97	3.22	^oxo^G_4_G_5_	3.44	3.12	^oxo^G_4_G_5_	3.40	3.42
**RcA-DNA**			**3G^oxo^-RcA-DNA**			**5G^oxo^-RcA-DNA**		
G_2_A_3_	1.99	2.11	G_2_A_3_	0.99	0.89	^oxo^G_2_A_3_	2.05	1.86
(5′*R*)cA_3_G_4_	2.00	5.03	(5′*R*)cA_3_G_4_^oxo^	3.27	3.13	(5′*R*)cA_3_G_4_	4.98	3.34
^oxo^G_4_G_5_	2.81	2.99	^oxo^G_4_G_5_	1.86	1.99	^oxo^G_4_G_5_	2.89	0.94

**Table 4:** Stacking energy interaction in kcal/mol within base pairs dimers of the discussed *ds*-oligonucleotides in their neutral (Neut.) and vertical neutral (after electron adoption by adiabatic radical cation) (VER.N) form geometries.

*ds*-DNA Stacking Energy (kcal/mol)								
**N-DNA**			**3G^oxo^-N-DNA**			**5G^oxo^-N-DNA**		
	**NEUT.**	**VER.N**		**NEUT.**	**VER.N**		**NEUT.**	**VER.N**
G_2_A_3_	−14.56	−14.55	G_2_A_3_	−14.23	−14.91	G_2_^oxo^A_3_	−14.43	−13.75
A_3_G_4_	−13.59	−14.88	A_3_G_4_^oxo^	−14.69	−14.63	A_3_G_4_	−13.38	−14.55
G_4_G_5_	−12.05	−13.39	G_4_^oxo^G_5_	−12.86	−13.03	G_4_G_5_	−12.09	−12.76
**ScA-DNA**			**3G^oxo^-ScA-DNA**			**5G^oxo^-ScA-DNA**		
G_2_A_3_	−12.94	−13.10	G_2_A_3_	−12.90	−13.10	^oxo^ G_2_A_3_	−12.82	−12.31
(5′*S*)cA_3_G_4_	−11.25	−11.20	(5′*S*)cA_3_G_4_^oxo^	−11.88	−11.07	(5′*S*)cA_3_G_4_	−11.40	−11.90
^oxo^G_4_G_5_	−14.15	−13.27	^oxo^G_4_G_5_	−14.68	−13.86	^oxo^G_4_G_5_	−14.16	−14.25
**RcA-DNA**			**3G^oxo^-RcA-DNA**			**5G^oxo^-RcA-DNA**		
G_2_A_3_	−13.23	−13.39	G_2_A_3_	−12.92	−12.86	^oxo^G_2_A_3_	−13.05	−10.58
(5′*R*)cA_3_G_4_	−13.39	−12.52	(5′*R*)cA_3_G_4_^oxo^	−14.73	−13.77	(5′*R*)cA_3_G_4_	−13.24	−13.70
^oxo^G_4_G_5_	−13.50	−12.92	^oxo^G_4_G_5_	−14.84	−14.28	^oxo^G_4_G_5_	−13.48	−14.54

**Table 5:** Hydrogen bond energy in kcal/mol of base pairs included in the structure of the discussed *ds*-oligonucleotides in their neutral (Neut.) and vertical neutral (after electron adoption by adiabatic radical cation) (VER.N) form: (a) calculated for an ideal base pair model, (b) calculated for base pairs extracted/selected from 2lsf.pdb [45] and (c) 5iv1.pdb [46] structures.

*ds*-DNA Hydrogen Bond Energy								
**N-DNA**			**3G^oxo^-N-DNA**			**5G^oxo^-N-DNA**		
	**NEUT.**	**VER.N**		**NEUT.**	**VER.N**		**NEUT.**	**VER.N**
G_2_C_2_	−17.23 −17.54 ^(a)^ −14.36 ^(b)^	−17.22 −18.10 ^(a)^	G_2_C_2_	−17.32	−17.36	^oxo^G_2_C_2_	−17.69	−18.16
A_3_T_3_	−10.81 −10.95 ^(a)^ −8.64 ^(b)^	−10.74 −9.75 ^(a)^	A_3_T_3_	−10.53	−10.44	A_3_T_3_	−10.80	−10.39
G_4_C_4_	−17.20	−17.00	^oxo^G_4_C_4_	−17.74 −18.04 ^(a)^ −16.83 ^(c)^	−17.97 −18.49 ^(a)^	G_4_C_4_	−17.26	−17.14
G_5_C_5_	−17.21	−17.73	G_5_C_5_	−17.23	−17.32	G_5_C_5_	−17.23	−17.31
**ScA-DNA**			**3G^oxo^-ScA-DNA**			**5G^oxo^-ScA-DNA**		
G_2_C_2_	−17.30	−17.48	G_2_C_2_	−17.25	−17.30	^oxo^G_2_C_2_	−17.84	−18.38
(5′*S*)cA_3_T_3_	−10.62 −10.98 ^(a)^ −5.89 ^(b)^	−10.81 −9.77 ^(a)^	(5′*S*)A_3_T_3_	−10.54	−10.61	(5′*S*)A_3_T_3_	−10.66	−9.99
G_4_C_4_	−17.10	−17.80	^oxo^G_4_C_4_	−17.58	−18.07	G_4_C_4_	−17.12	−17.04
G_5_C_5_	−17.06	−17.19	G_5_C_5_	−17.08	−17.23	G_5_C_5_	−17.04	−17.06
**RcA-DNA**			**3G^oxo^-RcA-DNA**			**5G^oxo^-RcA-DNA**		
G_2_C_2_	−17.19	−17.15	G_2_C_2_	−16.94	−17.03	^oxo^G_2_C_2_	−17.81	−18.01
(5′*R*)cA_3_T_3_	−10.60 −10.98 ^(a)^	−10.49 −9.74 ^(a)^	(5′*R*)A_3_T_3_	−10.65	−10.55	(5′*R*)A_3_T_3_	−10.65	−9.88
G_4_C_4_	−16.77	−17.86	^oxo^G_4_C_4_	−18.07	−18.60	G_4_C_4_	−16.82	−17.50
G_5_C_5_	−16.95	−17.17	G_5_C_5_	−16.81	−16.85	G_5_C_5_	−16.96	−16.80

**Table 6:** Electronic properties in eV: Vertical (VIP) and adiabatic ionization potential (AIP) of the discussed double-stranded trimers, dimers, as well as single base pairs isolated from their parent *ds*-tetramers, calculated at the M062x/6-31+G** level of theory in the aqueous phase.

	AIP	VIP			AIP	VIP		AIP	VIP
***ds*-trimers**									
**N-DNA**			**3G^oxo^-N-DNA**				**5G^oxo^-N-DNA**		
**G_2_A_3_G_4_**	5.72	6.10	**G_2_A_3_^oxo^G_4_**		5.51	5.85	**^oxo^G_2_A_3_G_4_**	5.45	5.90
**A_3_G_4_G_5_**	5.64	6.03	**A_3_^oxo^G_4_G_5_**		5.37	5.79	**A_3_G_4_G_5_**	6.01	6.02
**ScA-DNA**			**3G^oxo^-ScA-DNA**				**5G^oxo^-ScA-DNA**		
**G_2_(5′*S*)cA_3_G_4_**	5.74	6.13	**G_2_(5′*S*)cA_3_^oxo^G_4_**		5.51	5.91	**^oxo^G_2_(5′*S*)cA_3_G_4_**	5.44	5.86
**(5′*S*)cA_3_G_4_G_5_**	5.69	6.08	**(5′*S*)cA_3_^oxo^G_4_G_5_**		5.45	5.88	**(5′*S*)cA_3_G_4_G_5_**	6.09	6.14
**RcA-DNA**			**3G^oxo^-RcA-DNA**				**5G^oxo^-RcA-DNA**		
**G_2_(5′*R*)cA_3_G_4_**	5.72	6.08	**G_2_(5′*R*)cA_3_^oxo^G_4_**		5.50	5.94	**^oxo^G_2_(5′*R*)cA_3_G_4_**	5.43	5.87
**(5′*R*)cA_3_G_4_G_5_**	5.66	6.03	**(5′*R*)cA_3_^oxo^G_4_G_5_**		5.47	5.91	**(5′*R*)cA_3_G_4_G_5_**	6.13	6.03
***ds*-dimers**									
**N-DNA**			**3G^oxo^-N-DNA**				**5G^oxo^-N-DNA**		
**G_2_A_3_**	6.13	6.15	**G_2_A_3_**		6.13	6.12	**^oxo^G_2_A_3_**	5.50	5.91
**A_3_G_4_**	5.73	6.12	**A_3_^oxo^G_4_**		5.48	5.88	**A_3_G_4_**	6.12	6.11
**G_4_G_5_**	5.68	6.05	**^oxo^G_4_G_5_**		5.40	5.83	**G_4_G_5_**	6.04	6.05
**ScA-DNA**			**3G^oxo^-ScA-DNA**				**5G^oxo^-ScA-DNA**		
**G_2_A_3_**	6.12	6.10	**G_2_A_3_**		6.13	6.10	**^oxo^G_2_A_3_**	5.47	5.87
**(5′*S*)cA_3_G_4_**	5.75	6.14	**(5′*S*)cA_3_G_4_**		5.52	5.93	**(5′*S*)cA_3_G_4_**	6.20	6.15
**G_4_G_5_**	5.78	6.12	**G_4_G_5_**		5.48	5.89	**G_4_G_5_**	6.13	6.13
**RcA-DNA**			**3G^oxo^-RcA-DNA**				**5G^oxo^-RcA-DNA**		
**G_2_A_3_**	6.12	6.10	**G_2_A_3_**		6.14	6.12	**^oxo^G_2_A_3_**	5.53	5.89
**(5′*R*)cA_3_G_4_**	5.74	6.09	**(5′*R*)cA_3_G_4_**		5.51	5.94	**(5′*R*)cA_3_G_4_**	6.13	6.10
**G_4_G_5_**	5.73	6.21	**G_4_G_5_**		5.49	5.93	**G_4_G_5_**	6.21	6.21
**Single base pairs**									
**N-DNA**			**3G^oxo^-N-DNA**				**5G^oxo^-N-DNA**		
**G_2_C_2_**	6.17	6.17	**G_2_C_2_**		6.19	6.19	**^oxo^G_2_C_2_**	**5.55**	**5.93**
**A_3_T_3_**	6.63	6.65	**A_3_T_3_**		6.69	6.65	**A_3_T_3_**	6.65	6.64
**G_4_C_4_**	**5.86**	**6.13**	**^oxo^G_4_C_4_**		**5.55**	**5.91**	**G_4_C_4_**	6.14	6.13
**G_5_C_5_**	6.15	6.20	**G_5_C_5_**		6.14	6.18	**G_5_C_5_**	6.19	6.20
**ScA-DNA**			**3G^oxo^-ScA-DNA**				**5G^oxo^-ScA-DNA**		
**G_2_C_2_**	6.13	6.19	**G_2_C_2_**		6.14	6.15	**^oxo^G_2_C_2_**	**5.55**	**5.92**
**(5′*S*)cA_3_T_3_**	6.65	6.68	**(5′*S*)cA_3_T_3_**		6.67	6.68	**(5′*S*)cA_3_T_3_**	6.83	6.69
**G_4_C_4_**	**5.84**	**6.14**	**^oxo^G_4_C_4_**		**5.55**	**5.94**	**G_4_C_4_**	6.19	6.18
**G_5_C_5_**	6.19	6.22	**G_5_C_5_**		6.19	6.22	**G_5_C_5_**	6.22	6.23
**RcA-DNA**			**3G^oxo^-RcA-DNA**				**5G^oxo^-RcA-DNA**		
**G_2_C_2_**	6.12	6.23	**G_2_C_2_**		6.16	6.18	**^oxo^G_2_C_2_**	**5.58**	**5.93**
**(5′*R*)c A_3_T_3_**	6.68	6.61	**(5′*R*)cA_3_T_3_**		6.65	6.61	**(5′*R*)cA_3_T_3_**	6.77	6.61
**G_4_C_4_**	**5.80**	**6.12**	**^oxo^G_4_C_4_**		**5.53**	**5.96**	**G_4_C_4_**	6.08	6.20
**G_5_C_5_**	6.18	6.22	**G_5_C_5_**		6.15	6.19	**G_5_C_5_**	6.19	6.22
**Ideal Base Pair Model**							**Isolated from 5ivl.pdb** [45] **and 21sf.pdb** [44] **Structure**		
	**AIP**			**VIP**					
**G:::C**	5.58			6.13				VIP	
**G^oxo^:::C**	5.55			5.90			G:::C^**^	6.14	
**ScA::T**	6.35			6.62			G^oxo^:::C^*^	5.82	
**RcA::T**	6.35			6.62			ScA::T^**^	6.71	
**A::T**	6.34			6.62			A::T^**^	6.66	

**Table 7:** RMSD (root-mean-square deviation) in Å of the atomic positions calculated for *ds*-DNAs in neutral and cation radical forms.

*ds*-oligo	Backbone	Bases	All Nucleic Acid
N-DNA	1.347	1.039	1.197
3G^oxo^-N-DNA	0.299	0.225	0.261
5G^oxo^-N-DNA	0.359	0.319	0.337
ScA-DNA	0.699	0.193	0.516
3G^oxo^-ScA-DNA	0.203	0.176	0.190
5G^oxo^-ScA-DNA	0.247	0.214	0.231
RcA-DNA	0.695	0.298	0.537
3G^oxo^-RcA-DNA	0.119	0.082	0.103
5G^oxo^-RcA-DNA	1.032	0.817	0.931

**Table 8:** Hirshfeld charge (Q) and spin (S) in [au] distribution in the shape of *ds*-oligonucleotides only nucleosides bases were taken into consideration, calculated at the M062X/D95*//M062x/6-31+G** level of theory in the aqueous phase. A—neutral, VC—vertical cation, C—adiabatic cation.

*ds*-DNA Hirshfeld Charge and Spin Density Population																	
**N-DNA**						**3G^oxo^-N-DNA**						**5G^oxo^-N-DNA**					
	**N**	**VC**		**C**			**N**	**VC**		**C**			**N**	**VC**		**C**	
	**Q**	**Q**	**S**	**Q**	**S**		**Q**	**Q**	**S**	**Q**	**S**		**Q**	**Q**	**S**	**Q**	**S**
**T_6_**	−0.05	−0.05		−0.04		**T_6_**	−0.05	−0.05		−0.04		**T_6_**	−0.05	−0.05		−0.05	
**C_5_**	0.18	0.19		0.18		**C_5_**	0.17	0.17		0.17		**C_5_**	0.18	0.18		0.17	
**C_4_**	0.19	0.22		0.32		**C_4_**	0.21	0.24		0.29		**C_4_**	0.19	0.19		0.20	
**T_3_**	−0.07	−0.05		−0.02		**T_3_**	−0.07	−0.05		−0.05		**T_3_**	−0.06	−0.06		−0.05	
**C_2_**	0.21	0.22		0.22		**C_2_**	0.20	0.21		0.21		**C_2_**	0.21	0.25		0.31	
**T_1_**	−0.08	−0.07		−0.07		**T_1_**	−0.08	−0.07		−0.07		**T_1_**	−0.08	−0.05		−0.02	
**A_1_**	0.01	0.01		0.00		**A_1_**	0.01	0.01		0.01		**A_1_**	0.02	0.06	0.01	0.05	0.02
**G_2_**	−0.14	−0.13		−0.14		**G_2_**	−0.14	−0.13		−0.13		**G_2_^oxo^**	−0.16	0.69	0.97	0.59	0.97
**A_3_**	0.03	0.06	0.02	0.05	0.03	**A_3_**	0.03	0.07	0.02	0.07	0.02	**A_3_**	0.03	0.06	0.02	0.05	0.01
**G_4_**	−0.18	0.67	0.96	0.55	0.96	**G_4_^oxo^**	−0.19	0.66	0.97	0.59	0.97	**G_4_**	−0.18	−0.17		−0.16	
**G_5_**	−0.14	−0.10	0.02	−0.09	0.02	**G_5_**	−0.13	−0.10	0.01	−0.10	0.01	**G_5_**	−0.14	−0.14		−0.13	
**A_6_**	0.03	0.04		0.03		**A_6_**	0.04	0.04		0.05		**A_6_**	0.04	0.04		0.04	
**ScA-DNA**						**3G^oxo^-ScA-DNA**						**5G^oxo^-ScA-DNA**					
**T_6_**	−0.06	−0.06		−0.05		**T_6_**	−0.06	−0.06		−0.05		**T_6_**	−0.06	−0.06		−0.06	
**C_5_**	0.21	0.22		0.22		**C_5_**	0.21	0.22		0.22		**C_5_**	0.21	0.21		0.22	
**C_4_**	0.13	0.16		0.23		**C_4_**	0.13	0.16		0.23		**C_4_**	0.12	0.12		0.13	
**T_3_**	−0.08	−0.07		−0.06		**T_3_**	−0.08	−0.07		−0.07		**T_3_**	−0.08	−0.07		−0.07	
**C_2_**	0.22	0.22		0.23		**C_2_**	0.22	0.22		0.23		**C_2_**	0.22	0.25		0.31	
**T_1_**	−0.07	−0.07		−0.05		**T_1_**	−0.06	−0.06		−0.06		**T_1_**	−0.05	−0.03		−0.01	
**A_1_**	0.00	0.00		−0.01		**A_1_**	−0.01	−0.01		−0.01		**A_1_**	0.06	0.11	0.02	0.10	
**G_2_**	−0.16	−0.16		−0.15		**G_2_**	−0.16	−0.16		−0.14		**G_2_^oxo^**	−0.24	0.63	0.97	0.53	0.97
**ScA_3_**	0.04	0.08	0.03	0.07	0.03	**ScA_3_**	0.03	0.07	0.03	0.07	0.02	**ScA_3_**	0.03	0.06	0.01	0.06	0.03
**G_4_**	−0.15	0.72	0.96	0.62	0.96	**G_4_^oxo^**	−0.16	0.71	0.96	0.61	0.97	**G_4_**	−0.14	−0.14		−0.13	
**G_5_**	−0.11	−0.08	0.01	−0.09	0.01	**G_5_**	−0.11	−0.08	0.01	−0.08	0.01	**G_5_**	−0.11	−0.11		−0.11	
**A_6_**	0.03	0.04		0.04		**A_6_**	0.03	0.04		0.04		**A_6_**	0.03	0.03		0.03	
**RcA-DNA**						**3G^oxo^-RcA-DNA**						**5G^oxo^-RcA-DNA**					
**T_6_**	0.05	−0.05		−0.05		**T_6_**	−0.05	−0.05		−0.05		**T_6_**	−0.05	−0.05		−0.05	
**C_5_**	−0.21	0.21		0.22		**C_5_**	0.21	0.22		0.23		**C_5_**	0.21	0.21		0.21	
**C_4_**	0.13	0.16		0.24		**C_4_**	0.14	0.17		0.23		**C_4_**	0.13	0.13		0.14	
**T_3_**	−0.06	−0.04		−0.04		**T_3_**	−0.09	−0.08		−0.08		**T_3_**	−0.06	−0.05		−0.08	
**C_2_**	0.22	0.23		0.23		**C_2_**	0.21	0.21		0.21		**C_2_**	0.23	0.26		0.30	
**T_1_**	−0.03	−0.03		−0.03		**T_1_**	−0.03	−0.03		−0.03		**T_1_**	−0.04	−0.02		0.00	
**A_1_**	−0.02	−0.02		−0.02		**A_1_**	−0.02	−0.02		−0.02		**A_1_**	−0.01	0.06	0.04	0.07	0.05
**G_2_**	−0.15	−0.15		−0.14		**G_2_**	−0.15	−0.14		−0.13		**G_2_^oxo^**	−0.17	0.66	0.95	0.55	0.92
**RcA_3_**	0.02	0.10	0.07	0.06	0.03	**RcA_3_**	0.06	0.10	0.02	0.08	0.02	**RcA_3_**	0.03	0.06	0.01	0.06	0.03
**G_4_**	−0.17	0.63	0.89	0.59	0.95	**G_4_^oxo^**	−0.19	0.67	0.96	0.61	0.97	**G_4_**	−0.16	−0.16		−0.13	
**G_5_**	−0.12	−0.07	0.04	−0.09	0.02	**G_5_**	−0.12	−0.08	0.02	−0.09	0.01	**G_5_**	−0.12	−0.12		−0.12	
**A_6_**	0.03	0.04		0.04		**A_6_**	0.04	0.04		0.05		**A_6_**	0.03	0.03		0.04	

**Table 9:** Energy barriers (in eV) for radical cation migration between base pairs within trimers. The vertical modes, i.e., the energies of each base pair’s radical cation, were calculated for their neutral geometry. The adiabatic modes, i.e., the energies of each base pair’s radical cation, were calculated for their cation geometry. Arrows indicate the direction of the hole transfer from one base pair to another, e.g., A^+^ → G, calculated at the M062x/6-31+G** level of theory in the aqueous phase [42].

*ds*-oligo	Mode	Discussed Trimers					
		G_2_A_3_G_4_			A_3_G_4_ G_5_		
		G_2_→A_3_	A_3_→G_4_	G_2_→G_4_	A_3_→G_4_	G_4_→G_5_	A_3_→G_5_
**N-DNA**	**Vertical**	0.49	−0.49	−0.03	−0.49	0.73	−0.04
	**Adiabatic**	0.46	−0.77	−0.31	−0.77	0.29	−0.48
		**G_2_A_3_**	**A_3_** **←** **G_4_**	**G_2_** **←** **G_4_**	**A_3_** **←** **G_4_**	**G_4_** **←** **G_5_**	**A_3_** **←** **G_5_**
	**Vertical**	**−0.46**	1.18	0.31	1.18	**−0.01**	0.89
	**Adiabatic**	**−0.46**	0.77	0.31	0.77	**−0.29**	0.48
**3G^oxo^-N-DNA**		**G_2_**→**A_3_**	**A_3_**→ **^oxo^G_4_**	**G_2_**→ **^oxo^G_4_**	**A_3_**→ **^oxo^G_4_**	**^oxo^G_4_**→**G_5_**	**A_3_**→**G_5_**
	**Vertical**	0.46	−0.77	−0.27	−0.77	0.98	−0.16
	**Adiabatic**	0.50	−1.14	−0.64	−1.14	0.59	−0.54
		**G_2_←A_3_**	**A_3_←^oxo^G_4_**	**G_2_←^oxo^G_4_**	**A_3_←^oxo^G_4_**	**^oxo^G_4_←G_5_**	**A_3_←G_5_**
	**Vertical**	−0.49	1.44	0.64	1.44	−0.23	0.85
	**Adiabatic**	−0.50	1.14	0.64	1.14	−0.59	0.54
**5G^oxo^-N-DNA**		**^oxo^G_2_**→**A_3_**	**A_3_**→**G_4_**	**^oxo^G_2_**→**G_4_**	**A_3_**→**G_4_**	**G_4_**→**G_5_**	**A_3_**→**G_5_**
	**Vertical**	1.45	−0.50	0.61	−0.50	0.06	−0.45
	**Adiabatic**	1.10	−0.51	0.59	−0.51	0.05	−0.46
		**^oxo^G_2_←A_3_**	**A_3_←G_4_**	**^oxo^G_2_←G_4_**	**A_3_←G_4_**	**G_4_←G_5_**	**A_3_←G_5_**
	**Vertical**	−0.69	0.50	−0.18	0.50	−0.05	0.46
	**Adiabatic**	−1.10	0.51	−0.59	0.51	−0.05	0.46
**ScA-DNA**		**G_2_**→**ScA_3_**	**ScA_3_**→**G_4_**	**G_2_**→**G_4_**	**ScA_3_**→**G_4_**	**G_4_**→**G_5_**	**ScA_3_**→**G_5_**
	**Vertical**	0.55	−0.51	0.02	−0.51	0.71	−0.11
	**Adiabatic**	0.52	−0.82	−0.29	−0.82	0.35	−0.47
		**G_2_←ScA_3_**	**ScA_3_←G_4_**	**G_2_←G_4_**	**ScA_3_←G_4_**	**G_4_←G_5_**	**ScA_3_←G_5_**
	**Vertical**	−0.46	1.16	0.35	1.16	−0.05	0.82
	**Adiabatic**	−0.52	0.82	0.29	0.82	−0.35	0.47
**3G^oxo^-ScA-DNA**		**G_2_**→**ScA_3_**	**ScA_3_**→**^oxo^G_4_**	**G_2_**→**^oxo^G_4_**	**cSA_3_**→**^oxo^G_4_**	**^oxo^G_4_**→**G_5_**	**ScA_3_**→**G_5_**
	**Vertical**	0.55	−0.73	−0.20	−0.73	1.01	−0.11
	**Adiabatic**	0.53	−1.12	−0.59	−1.12	0.64	−0.48
		**G_2_←ScA_3_**	**ScA_3_←^oxo^G_4_**	**G_2_←^oxo^G_4_**	**ScA_3_←^oxo^G_4_**	**^oxo^G_4_←G_5_**	**ScA_3_←G_5_**
	**Vertical**	**−0.53**	1.47	0.60	1.47	**−0.25**	0.83
	**Adiabatic**	**−0.53**	1.12	0.59	1.12	**−0.64**	0.48
**5G^oxo^-ScA-DNA**		**^oxo^G_2_**→**ScA_3_**	**ScA_3_**→**G_4_**	**^oxo^G_2_**→**G_4_**	**ScA_3_**→**G_4_**	**G_4_**→**G_5_**	**ScA_3_**→**G_5_**
	**Vertical**	1.48	−0.61	0.67	−0.61	0.05	−0.59
	**Adiabatic**	1.28	−0.64	0.64	−0.64	0.03	−0.61
		**^oxo^G_2_←ScA_3_**	**ScA_3_←G_4_**	**^oxo^G_2_←G_4_**	**ScA_3_←G_4_**	**G_4_←G_5_**	**ScA_3_←G_5_**
	**Vertical**	−0.87	0.52	−0.23	0.52	−0.04	0.48
	**Adiabatic**	−1.28	0.64	−0.64	0.64	−0.03	0.61
**RcA-DNA**		**G_2_**→**RcA_3_**	**RcA_3_**→**G_4_**	**G_2_**→**G_4_**	**cA_3_**→**G_4_**	**G_4_**→**G_5_**	**cA_3_**→**G_5_**
	**Vertical**	0.49	−0.52	0.02	−0.52	0.69	−0.18
	**Adiabatic**	0.55	−0.88	−0.33	−0.88	0.38	−0.50
		**G_2_←RcA_3_**	**RcA_3_←G_4_**	**G_2_←G_4_**	**RcA_3_←G_4_**	**G_4_←G_5_**	**RcA_3_←G_5_**
	**Vertical**	−0.42	1.08	0.46	1.08	−0.06	0.70
	**Adiabatic**	−0.55	0.88	0.33	0.88	−0.38	0.50
**3G^oxo^-RcA-DNA**		**G_2_**→**RcA_3_**	**cA_3_**→**^oxo^G_4_**	**G_2_**→**G_4_**	**RcA_3_**→**^oxo^G_4_**	**^oxo^G_4_**→**G_5_**	**RcA_3_**→**G_5_**
	**Vertical**	0.45	−0.69	−0.19	−0.69	0.96	−0.16
	**Adiabatic**	0.50	−1.12	−0.63	−1.12	0.62	−0.50
		**G_2_←RcA_3_**	**RcA_3_←^oxo^G_4_**	**G_2_←^oxo^G_4_**	**RcA_3_←G_4_**	**^oxo^G_4_←G_5_**	**RcA_3_←G_5_**
	**Vertical**	−0.47	1.39	0.66	1.39	−0.20	0.76
	**Adiabatic**	−0.50	1.12	0.63	1.12	−0.62	0.50
**5G^oxo^-RcA-DNA**		**^oxo^G_2_**→**RcA_3_**	**RcA_3_**→**G_4_**	**^oxo^G_2_**→**G_4_**	**RcA_3_**→**G_4_**	**G_4_**→**G_5_**	**RcA_3_**→**G_5_**
	**Vertical**	1.36	−0.53	0.67	−0.53	0.08	−0.61
	**Adiabatic**	1.19	−0.69	−0.50	−0.69	0.11	−0.58
		**^oxo^G_2_←RcA_3_**	**RcA_3_←G_4_**	**^oxo^G_2_←G_4_**	**RcA_3_←G_4_**	**G_4_←G_5_**	**RcA_3_←G_5_**
	**Vertical**	−0.80	0.47	−0.11	0.47	0.01	0.36
	**Adiabatic**	−1.19	0.69	−0.50	0.69	−0.11	0.58

**Table 10:** Nuclear relaxation energy λ [eV] and hole transfer rate constant *k*_HT_ [s^−1^], energy barrier ΔG [eV], activation energy *E*_a_ [eV], and electron coupling energies *V_12_* [eV] of hole transfer between base pairs, calculated at the M062x/6-31+G** level of theory in the aqueous phase ([42] and references cited therein).

**System**	***ds*-DNA Hole Transfer between Stacked Base Pairs**																
	λ	ΔG	*E*_a_	*V*_12_	*K*_HT_		λ	ΔG	*E*_a_	*V*_12_	*K*_HT_		λ	ΔG	*E*_a_	*V*_12_	*k*_HT_
	**N-DNA**					**3G^oxo^-N-DNA**						**5G^oxo^-N-DNA**					
**G_2_←A_3_**	0.00	−0.46	18.60	0.221	0.00	**G_2_←A_3_**	0.01	−0.50	10.09	0.220	0.00	**^oxo^G_2_←A_3_**	0.41	−1.10	0.29	0.320	3.2 × 10^10^
**A_3_→G_4_**	0.28	−0.77	0.22	0.246	3.8 × 10^11^	**A_3_→^oxo^G_4_**	0.37	−1.14	0.41	0.363	5.2 × 10^8^	**A_3_→G_4_**	0.01	−0.51	4.31	0.246	0.00
**G_4_←G_5_**	0.44	−0.29	0.01	0.051	4.0 × 10^13^	**^oxo^G_4_←G_5_**	0.38	−0.59	0.03	0.113	1.1 × 10^14^	**G_4_←G_5_**	0.02	−0.05	0.01	0.048	1.7 × 10^14^
	**ScA-DNA**					**3G^oxo^-ScA-DNA**						**5G^oxo^-ScA-DNA**					
**G_2_←ScA_3_**	0.06	−0.52	0.86	0.263	14.58	**G_2_←cSA_3_**	0.02	−0.53	4.30	0.271	0.00	**^oxo^G_2_←ScA_3_**	0.41	−1.28	0.45	0.367	7.3 × 10^7^
**ScA_3_→G_4_**	0.31	−0.82	0.21	0.264	6.4 × 10^11^	**ScA_3_→^oxo^G_4_**	0.39	−1.12	0.35	0.378	5.5 × 10^9^	**ScA_3_→G_4_**	0.04	−0.64	2.62	0.271	0.00
**G_4_←G_5_**	0.36	−0.35	0.00	0.035	3.4 × 10^13^	**^oxo^G_4_←G_5_**	0.37	−0.64	0.05	0.157	1.0 × 10^14^	**G_4_←G_5_**	0.01	−0.03	0.01	0.038	1.5 × 10^14^
	**RcA-DNA**					**3G^oxo^-RcA-DNA**						**5G^oxo^-RcA-DNA**					
**G_2_←RcA_3_**	0.13	−0.55	0.33	0.260	9.1 × 10^9^	**G_2_←RcA_3_**	0.03	−0.50	1.91	0.268	0.00	**^oxo^G_2_←RcA_3_**	0.39	−1.19	0.41	0.352	3.28 × 10^8^
**RcA_3_→G_4_**	0.35	−0.88	0.19	0.297	1.3 × 10^12^	**RcA_3_→^oxo^G_4_**	0.43	−1.12	0.27	0.349	7.8 × 10^10^	**RcA_3_→G_4_**	0.16	−0.69	0.43	0.291	1.75 × 10^8^
**G_4_←G_5_**	0.31	−0.38	0.00	0.086	1.9 × 10^14^	**^oxo^G_4_←G_5_**	0.34	−0.62	0.06	0.131	5.3 × 10^13^	**G_4_←G_5_**	0.12	−0.11	0.00	0.085	3.5 × 10^14^

## Supplementary Materials

The following materials can be online: **Table S1.** The energies (in Hartree) of Neural, Vertical Cation, Adiabatic Cation and Vertical Neutral forms of ideal base pairs, base pairs extracted from 2lsf.pdb [1] and 5iv1.pdb [2] files calculated at the M062x/6-31+G** level of theory in the aqueous phase. **Table S2.** The energies (in Hartree) of Neural. Vertical Cation. Adiabatic Cation and Vertical Neutral forms of base pairs extracted from *ds*-oligonucleotides calculated at the M062x/6-31+G** level of theory in the aqueous phase. **Table S3.** The energies (in Hartree) of Neural. Vertical Cation. Adiabatic Cation and Vertical Neutral forms of base pairs dimers extracted from *ds*-oligonucleotides calculated at the M062x/6-31+G** level of theory in the aqueous phase. **Table S4.** Energies (in Hartree) of Neutral. Vertical Cation. Adiabatic Cation and Vertical Neutral forms of *ds*-trimers extracted from *ds*-oligonucleotides calculated at the M062x/6-31+G** level of theory in the aqueous phase. **Table S5.** The Ground and Excitation state energies and Excitation and HOMO Energies as well as corresponding Dipole Moments (Ground Excitation and Transition) of base pair dimers extracted from *ds*-oligonucleotides. calculated at the M062x/6-31+G** level of theory in the aqueous phase using the DFT or TD-DFT methodology. **Eighteen pdb files of discussed *ds*-oligo structures:** ScA_DNA_Neutral.pdb, ScA_DNA_Cation.pdb, RcA_DNA_Neutral, RcA_DNA_Cation.pdb, N_DNA_Neutral.pdb, N_DNA_Cation.pdb, 5Goxo_ScA_DNA_Neutral.pdb, 5Goxo_ScA_DNA_Cation.pdb, 5Goxo_RcA_DNA_Neutral.pdb, 5Goxo_RcA_DNA_Cation.pdb, 5Goxo_N_DNA_Neutral.pdb, 5Goxo_N_DNA_Cation.pdb, 3Goxo_ScA_DNA_Neutral.pdb, 3Goxo_ScA_DNA_Cation.pdb, 3Goxo_RcA_DNA_Neutral.pdb, 3Goxo_RcA_DNA_Cation.pdb, 3Goxo_N_DNA_Neutral.pdb, 3Goxo_N_DNA_Cation.pdb.
