# Supplementary material for: The Influence of Single, Tandem, and Clustered DNA Damage on the Electronic Properties of the Double Helix: A Theoretical Study
Source: Molecules. 2020 Jul 8;25(14):3126. doi: 10.3390/molecules25143126 (PMC7397046; doi:10.3390/molecules25143126)
Supplement: Supplementary file 1 [file molecules-25-03126-s001.pdf]

molecules

Supplementary Materials

The Influence of Single, Tandem and Clustered DNA Damage on The Electronic Properties of the Double Helix. A Theoretical Study.

Boleslaw T. Karwowski *

Department of Biopharmacy, Medical University of Lodz, Muszynskiego Street 1, 90-151 Lodz, Poland
* Correspondence: Boleslaw.Karwowski@umed.lodz.pl

Received: 13 June 2020; Accepted: 5 July 2020; Published: date

Abstract: Oxidatively generated damage to DNA frequently appears in the human genome as the effect of aerobic metabolism or as the result of exposure to exogenous oxidizing agents such as ionization radiation. In this paper, the electronic properties of single, tandem and clustered DNA damage in comparison with native *ds*-DNA are discussed as a comparative analysis for the first time. A single lesion—8-oxo-7,8-dihydro-2′-deoxyguanosine (G^{oxo}), a tandem lesion—(5′*S*) and (5′*R*) 5′,8-cyclo-2′-deoxyadenosine (cdA), and the presence of both of them in one helix turn as clustered DNA damage were chosen and taken into consideration. The lowest vertical and adiabatic potential (VIP ~ 5.9 and AIP ~ 5.5 eV, respectively) were found for G^{oxo}, independently of the discussed DNA lesion type and their distribution within the double helix. Moreover, the VIP and AIP were assigned for *ds*-trimers, *ds*- dimers and single base pairs isolated from parental *ds*-hexamers in their neutral and cationic forms. The above results were confirmed by the charge and spin density population which revealed that G^{oxo} can be considered as a cation radical point of destination independently of the DNA damage type (single, tandem or clustered). Additionally, the different influences of cdA on the charge transfer rate were found and discussed in the context of tandem and clustered lesions. Because oligonucleotide lesions are effectively produced as a result of ionization factors, the presented data in this article might be valuable in developing a new scheme of anticancer radiotherapy efficiency.

Keywords: DNA damage; electronic properties; charge transfer; DFT; (5′*R*)/(5′*S*)-5′,8-cyclo-2′-deoxyadenosine; 8-oxo-7,8-dihydro-2′-deoxyguanosine

Table S1. The energies (in Hartree) of Neural, Vertical Cation, Adiabatic Cation and Vertical Neutral forms of ideal base pairs, base pairs extracted from ***2lsf.pdb [1]** and ****5iv1.pdb [2]** files calculated at the M062x/6-31+G** level of theory in the aqueous phase.

Base Pairs	Neural	Vertical Cation	Adiabatic Cation	Vertical Neutral
**Ideal model of Base pairs**				
**A::T**	-921.1939648	-920.9505955	-920.9607985	-921.1826007
**G:::C**	-937.2537902	-937.0285732	-937.0400476	-937.2416197
**^{oxo}G:::C**	-1012.479573	-1012.262807	-1012.2757625	-1012.466325
**(5'R)cA::T**	-921.1935204	-920.9503885	-920.9603189	-921.1815302
**(5'S)cA::T**	-921.1935113	-920.9501698	-920.9603045	-921.1818484
**Base pairs structure obtained from 2lsf.pdb and 5iv1.pdb flies**				
**A::T ***	-921.1840142	-920.939105		
**G:::C ***	-937.2420589	-937.0163676		
**^{oxo}G:::C ****	-1012.447201	-1012.233256		
**(5'S)cA::T ***	-921.1789845	-920.9325337		

1) Zaliznyak, T.; Lukin, M.; De Los Santos, C. Structure and stability of duplex DNA containing (5′ S)-5′,8-cyclo-2′-deoxyadenosine: An oxidatively generated lesion repaired by NER. *Chem. Res. Toxicol.* **2012**, *25*, 2103–2111.
2) Hoppins, J.J.; Gruber, D.R.; Miears, H.L.; Kiryutin, A.S.; Kasymov, R.D.; Petrova, D. V.; Endutkin, D. V.; Popov, A. V.; Yurkovskaya, A. V.; Fedechkin, S.O.; et al. 8-oxoguanine affects DNA backbone conformation in the EcoRI recognition site and inhibits its cleavage by the enzyme. *PLoS One* **2016**, *11*, 1–15.

Table S2. The energies (in Hartree) of Neural. Vertical Cation. Adiabatic Cation and Vertical Neutral forms of base pairs extracted from *ds*-oligonucleotides calculated at the M062x/6-31+G** level of theory in the aqueous phase.

Base Pairs	Neural	Vertical Cation	Adiabatic Cation	Vertical Neutral
**N-DNA**				
G_2C_2	-937.2515365	-937.0248069	-937.0248172	-937.251061
A_3T_3	-921.1914685	-920.9472501	-920.9478538	-921.1913757
G_4C_4	-937.2506957	-937.0253013	-937.0354166	-937.2361792
G_5C_5	-937.251706	-937.0239395	-937.0256507	-937.2513379
**$3G^{oxo}$-N-DNA**				
G_2C_2	-937.2518086	-937.0244205	-937.0244229	-937.2518063
A_3T_3	-921.1910684	-920.9466478	-920.9453924	-921.1908506
$^{oxo}G_4C_4$	-1012.47661	-1012.259452	-1012.272683	-1012.46401
G_5C_5	-937.2517046	-937.0244585	-937.0259489	-937.2515679
**$5G^{oxo}$-N-DNA**				
$^{oxo}G_2C_2$	-1012.477392	-1012.259391	-1012.273612	-1012.464258
A_3T_3	-921.1915975	-920.9477029	-920.9472794	-921.1907142
G_4C_4	-937.2510816	-937.0258433	-937.025483	-937.2508351
G_5C_5	-937.2517442	-937.0240696	-937.0244155	-937.2515767
**ScA-DNA**				
G_2C_2	-937.2511892	-937.0238358	-937.0259761	-937.2510604
$(5'S)cA_3T_3$	-921.1905969	-920.9451698	-920.9461375	-921.1904555
G_4C_4	-937.2507892	-937.025097	-937.0362833	-937.2389462
G_5C_5	-937.2509798	-937.0222665	-937.023687	-937.251174
**$3G^{oxo}$-ScA-DNA**				
G_2C_2	-937.2510038	-937.0248767	-937.0253665	-937.2508968
$(5'S)cA_3T_3$	-921.1897992	-920.9441954	-920.9445773	-921.1897172
$^{oxo}G_4C_4$	-1012.476217	-1012.258011	-1012.272207	-1012.463747
G_5C_5	-937.2512452	-937.0226462	-937.0237979	-937.2513099
**$5G^{oxo}$-ScA-DNA**				
$^{oxo}G_2C_2$	-921.1904198	-920.9444135	-920.9394683	-921.1887428
$(5'S)cA_3T_3$	-937.2508544	-937.0238309	-937.0234412	-937.2504929
G_4C_4	-937.2511049	-937.0223062	-937.0224506	-937.2510829
G_5C_5	-921.1904198	-920.9444135	-920.9394683	-921.1887428
**RcA-DNA**				
G_2C_2	-937.25062	-937.0215919	-937.025336	-937.25025
$(5'R)cA_3T_3$	-921.1904662	-920.947387	-920.9450889	-921.1893736
G_4C_4	-937.249566	-937.0245176	-937.0363958	-937.2396484
G_5C_5	-937.250898	-937.0222202	-937.0237399	-937.2510176
**$3G^{oxo}$-RcA-DNA**				
$^{oxo}G_2C_2$	-937.2505518	-937.023535	-937.0242608	-937.2505971
$(5'R)cA_3T_3$	-921.1899409	-920.9469424	-920.9454265	-921.189615
G_4C_4	-1012.476589	-1012.257676	-1012.273317	-1012.465407
G_5C_5	-937.250907	-937.0234679	-937.024811	-937.2509086
**$5G^{oxo}$-RcA-DNA**				
$^{oxo}G_2C_2$	-1012.476744	-1012.258971	-1012.271661	-1012.464499
$(5'R)cA_3T_3$	-921.1905944	-920.9476337	-920.9416463	-921.1889598
G_4C_4	-937.2495197	-937.0216263	-937.0259288	-937.2516054
G_5C_5	-937.2509963	-937.0224543	-937.0234582	-937.2509295

Table S3. The energies (in Hartree) of Neural. Vertical Cation. Adiabatic Cation and Vertical Neutral forms of base pairs dimmers extracted from *ds*-oligonucleotides calculated at the M062x/6-31+G** level of theory in the aqueous phase.

Base Pairs	Neural	Vertical Cation	Adiabatic Cation	Vertical Neutral
		**N-DNA**		
$[G_2A_3]^*$ $[T_3C_2]$	-1858.4662049	-1858.2402029	-1858.2410006	-1858.4656238
$[A_3G_4]^*$ $[C_4T_3]$	-1858.4638196	-1858.2390086	-1858.2533369	-1858.4512598
$[G_4G_5][C_5C_4]$	-1874.5215990	-1874.2991690	-1874.3128062	-1874.5088581
		**3G^{oxo}-N-DNA**		
$[G_2A_3]^*$ $[T_3C_2]$	-1858.4655520	-1858.2406567	-1858.2404284	-1858.4664199
$[A_3{}^{oxo}G_4]^*$ $[C_4T_3]$	-1933.6910878	-1933.4750033	-1933.4897700	-1933.6781688
$[{}^{oxo}G_4G_5][C_5C_4]$	-1949.7488019	-1949.5344716	-1949.5503885	-1949.7363481
		**5G^{oxo}-N-DNA**		
$[{}^{oxo}G_2A_3]^*$ $[T_3C_2]$	-1933.6919780	-1933.4746339	-1933.4896753	-1933.6768779
$[A_3G_4]^*$ $[C_4T_3]$	-1858.4639951	-1858.2395318	-1858.2391753	-1858.4647407
$[G_4G_5][C_5C_4]$	-1874.5220946	-1874.2997211	-1874.3000445	-1874.5227427
		**ScA-DNA**		
$[G_2(5'S)cA_3]^*$ $[T_3C_2]$	-1858.4624098	-1858.2381467	-1858.2376871	-1858.4623984
$[(5'S)cA_3G_4]^*$ $[C_4T_3]$	-1858.4593178	-1858.2337251	-1858.2478734	-1858.4472565
$[G_4G_5][C_5C_4]$	-1874.5243240	-1874.2993118	-1874.3120667	-1874.5112690
		**3G^{oxo}-ScA-DNA**		
$[G_2(5'S)cA_3]^*$ $[T_3C_2]$	-1858.4613578	-1858.2373413	-1858.2361334	-1858.4614963
$[(5'S)cA_3{}^{oxo}G_4]^*$ $[C_4T_3]$	-1933.6849432	-1933.4671262	-1933.4820212	-1933.6711063
$[{}^{oxo}G_4G_5][C_5C_4]$	-1949.7508578	-1949.5345269	-1949.5493523	-1949.7371512
		**5G^{oxo}-ScA-DNA**		
$[{}^{oxo}G_2(5'S)cA_3]^*$ $[T_3C_2]$	-1933.6878383	-1933.4720391	-1933.4869939	-1933.6728273
$[(5'S)c\ A_3G_4]^*$ $[C_4T_3]$	-1858.4594392	-1858.2336109	-1858.2317152	-1858.4582043
$[G_4G_5][C_5C_4]$	-1874.5245248	-1874.2993905	-1874.2991326	-1874.5242816
		**RcA-DNA**		
$[G_2(5'R)cA_3]^*$ $[T_3C_2]$	-1858.4621679	-1858.2380526	-1858.2371384	-1858.4609692
$[(5'R)cA_3G_4]^*$ $[C_4T_3]$	-1858.4613627	-1858.2373820	-1858.2505008	-1858.4489790
$[G_4G_5][C_5C_4]$	-1874.5219777	-1874.2937252	-1874.3115109	-1874.5112559
		**3G^{oxo}-RcA-DNA**		
$[G_2(5'R)cA_3]^*$ $[T_3C_2]$	-1858.4610836	-1858.2360537	-1858.2354004	-1858.4607075
$[(5'R)cA_3{}^{oxo}G_4]^*$ $[C_4T_3]$	-1933.6899995	-1933.4715353	-1933.4874127	-1933.6769688
$[{}^{oxo}G_4G_5][C_5C_4]$	-1949.7511472	-1949.5334067	-1949.5494469	-1949.7390795
		**5G^{oxo}-RcA-DNA**		
$[{}^{oxo}G_2(5'R)cA_3]^*$ $[T_3C_2]$	-1933.6881339	-1933.4718496	-1933.4849831	-1933.6703228
$[(5'R)c\ A_3G_4]^*$ $[C_4T_3]$	-1858.4612107	-1858.2370532	-1858.2358936	-1858.4623936
$[G_4G_5][C_5C_4]$	-1874.5220028	-1874.2939453	-1874.2972427	-1874.5256992

Table S4. Energies (in Hartree) of Neutral. Vertical Cation. Adiabatic Cation and Vertical Neutral forms of *ds*-trimmers extracted from *ds*-oligonucleotides calculated at the M062x/6-31+G** level of theory in the aqueous phase.

***ds*-Trimers**	**Neural**	**Vertical Cation**	**Adiabatic Cation**	**Vertical Neutral**
		**N-DNA**		
$G_2A_3G_4$	-2795.7392784	-2795.5151644	-2795.5292377	-2795.7261810
$A_3G_4G_5$	-2795.7356682	-2795.5140084	-2795.5282692	-2795.7237933
		**3G^{oxo}-N-DNA**		
$^{oxo}G_2A_3G_4$	-2870.9681367	-2870.7531569	-2870.7657917	-2870.9547305
$A_3{}^{oxo}G_4G_5$	-2870.9658623	-2870.7529526	-2870.7685942	-2870.9510353
		**5G^{oxo}-N-DNA**		
$G_2A_3{}^{oxo}G_4$	-2870.9655128	-2870.7487217	-2870.7651812	-2870.9516374
$A_3{}^{oxo}G_4G_5$	-2795.7359053	-2795.5146285	-2795.5149884	-2795.7377389
		**ScA-DNA**		
G_2(5'S)cA_3G_4	-2795.7324738	-2795.5072832	-2795.5216115	-2795.7204734
(5'S)c$A_3G_4G_5$	-2795.7337350	-2795.5101389	-2795.5247755	-2795.7203500
		**3G^{oxo}-ScA-DNA**		
$^{oxo}G_2$(5'S)cA_3G_4	-2870.9580179	-2870.7406566	-2870.7556405	-2870.9443845
(5'S)c$A_3{}^{oxo}G_4G_5$	-2870.9601261	-2870.7442006	-2870.7597812	-2870.9447815
		**5G^{oxo}-ScA-DNA**		
$^{oxo}G_2$(5'R)cA_3G_4	-2870.9577503	-2870.7422991	-2870.7579932	-2870.9431828
(5'R)c$A_3{}^{oxo}G_4G_5$	-2795.7340218	-2795.5084372	-2795.5101179	-2795.7330089
		**RcA-DNA**		
G_2(5'R)cA_3G_4	-2795.7340000	-2795.5106661	-2795.5238856	-2795.7216147
(5'R)c$A_3G_4G_5$	-2795.7347013	-2795.5131079	-2795.5267043	-2795.7211559
		**3G^{oxo}-RcA-DNA**		
$^{oxo}G_2$(5'R)cA_3G_4	-2870.9624727	-2870.7443499	-2870.7602276	-2870.9493532
(5'R)c$A_3{}^{oxo}G_4G_5$	-2870.9656531	-2870.7484273	-2870.7646531	-2869.5062831
		**5G^{oxo}-RcA-DNA**		
$^{oxo}G_2$(5'R)cA_3G_4	-2870.9596886	-2870.7438529	-2870.7601723	-2870.9446646
(5'R)c$A_3{}^{oxo}G_4G_5$	-2795.7346097	-2795.5129117	-2795.5093099	-2795.7375374

Table S5. The Ground and Excitation state energies and Excitation and HOMO Energies as well as corresponding Dipole Moments (Ground Excitation and Transition) of base pair dimers extracted from *ds*-oligonucleotides. calculated at the M062x/6-31+G** level of theory in the aqueous phase using the DFT or TD-DFT methodology.

Base Pairs Dimer	Energy of the state [Hartree]		Dipole moment [D]			Energies [eV]		
	Ground	Excitation	Ground	Excitation	Transition	HOMO	HOMO-1	Excitation
				N-DNA				
$[G_1A_3]^*[T_3C_2]$	-1858.4662049	-1858.3332274	9.23	8.47	0.96	-0.26140	-0.27950	4.8179
$[A_3G_4]^*[C_4T_3]$	-1858.4638196	-1858.3309487	10.41	12.36	0.87	-0.26141	-0.27946	4.8279
$[G_4G_5]^*[C_5C_4]$	-1874.5215990	-1874.3841340	14.82	13.50	0.21	-0.25872	-0.26243	4.8907
				$3G^{oxo}$-N-DNA				
$[G_1A_3]^*[T_3C_2]$	-1858.4655520	-1858.3327197	9.41	8.57	0.13	-0.261960	-0.278150	4.9048
$[A_3{}^{oxo}G_4]^*[C_4T_3]$	-1933.6910878	-1933.5651665	16.80	16.04	1.39	-0.25308	-0.27978	4.5143
$[{}^{oxo}G_4G_5]^*[C_5C_4]$	-1949.7488019	-1949.6216797	21.60	20.35	1.95	-0.25255	-0.26087	4.6006
				$5G^{oxo}$-N-DNA				
$[{}^{oxo}G_1A_3]^*[T_3C_2]$	-1933.6919780	-1933.5652791	15.87	14.19	1.94	-0.25525	-0.27876	4.6017
$[A_3G_4]^*[C_4T_3]$	-1858.4639951	-1858.3312489	10.57	12.44	0.82	-0.26113	-0.27923	4.8169
$[G_4G_5]^*[C_5C_4]$	-1874.5220946	-1874.3845818	14.81	13.59	0.21	-0.25876	-0.26231	4.8898
				ScA-DNA				
$[G_1(5'S)cA_3]^*[T_3C_2]$	-1858.4624098	-1858.3300622	9.77	8.54	1.23	-0.26179	-0.28112	4.9228
$[(5'S)cA_3G_4]^*[C_4T_3]$	-1858.4593178	-1858.3276373	9.97	9.93	1.08	-0.26317	-0.28259	4.8998
$[G_4G_5]^*[C_5C_4]$	-1874.5243240	-1874.3845262	14.24	14.17	0.26	-0.26240	-0.26495	4.8895
				$3G^{oxo}$-ScA-DNA				
$[G_1(5'S)cA_3]^*[T_3C_2]$	-1858.4613578	-1858.3292904	9.77	8.77	1.28	-0.26151	-0.28146	4.9222
$[(5'S)cA_3{}^{oxo}G_4]^*[C_4T_3]$	-1933.6849432	-1933.5593683	15.41	13.79	2.15	-0.25579	-0.28356	4.5524
$[{}^{oxo}G_4G_5]^*[C_5C_4]$	-1949.7508578	-1949.6251431	20.73	19.66	2.21	-0.25407	-0.2656	4.5760
				$5G^{oxo}$-ScA-DNA				
$[{}^{oxo}G_1(5'S)cA_3]^*[T_3C_2]$	-1933.6878383	-1933.5607935	15.90	14.90	2.42	-0.25428	-0.28126	4.5994
$[(5'S)cA_3G_4]^*[C_4T_3]$	-1858.4594392	-1858.3275632	9.85	9.83	1.02	-0.26342	-0.28333	4.8958
$[G_4G_5]^*[C_5C_4]$	-1874.5245248	-1874.3847722	14.15	13.99	0.22	-0.26246	-0.26522	4.8902
				RcA-DNA				
$[G_1(5'R)cA_3]^*[T_3C_2]$	-1858.4621679	-1858.3293333	8.92	9.92	2.09	-0.26113	-0.28021	4.9083
$[(5'R)cA_3G_4]^*[C_4T_3]$	-1858.4613627	-1858.3298090	9.83	9.41	0.88	-0.26017	-0.28196	4.7737
$[G_4G_5]^*[C_5C_4]$	-1874.5219777	-1874.3828555	14.10	12.72	0.04	-0.26080	-0.26711	4.8732
				$3G^{oxo}$-RcA-DNA				
$[G_1(5'R)cA_3]^*[T_3C_2]$	-1858.4610836	-1858.3277212	9.37	8.43	1.41	-0.26178	-0.28144	4.9188
$[(5'R)cA_3{}^{oxo}G_4]^*[C_4T_3]$	-1933.6899995	-1933.5620910	16.14	14.34	1.57	-0.25597	-0.28163	4.5961
$[{}^{oxo}G_4G_5]^*[C_5C_4]$	-1949.7511472	-1949.6230159	21.22	20.21	2.08	-0.25518	-0.26481	4.6256
				$5G^{oxo}$-RcA-DNA				
$[{}^{oxo}G_1(5'R)cA_3]^*[T_3C_2]$	-1933.6881339	-1933.5610793	15.52	13.57	2.98	-0.25451	-0.28035	4.6016
$[(5'R)cA_3G_4]^*[C_4T_3]$	-1858.4612107	-1858.3298347	9.73	9.32	0.84	-0.26051	-0.28189	4.7819
$[G_4G_5]^*[C_5C_4]$	-1874.5220028	-1874.3827669	14.15	12.55	0.05	-0.26062	-0.26688	4.8693

© 2020 by the authors. Submitted for possible open access publication under the terms and conditions of the Creative Commons Attribution (CC BY) license (http://creativecommons.org/licenses/by/4.0/).
